# Changes in Phenolic Compounds and Cellular Ultrastructure of Arctic and Antarctic Strains of *Zygnema* (Zygnematophyceae, Streptophyta) after Exposure to Experimentally Enhanced UV to PAR Ratio

**DOI:** 10.1007/s00248-012-0096-9

**Published:** 2012-08-18

**Authors:** Martina Pichrtová, Daniel Remias, Louise A. Lewis, Andreas Holzinger

**Affiliations:** 1 Department of Botany, Faculty of Science, Charles University in Prague, Benátská 2, 12801 Prague 2, Czech Republic; 2 Institute of Botany, Academy of Sciences of the Czech Republic, Dukelská 135, 37982 Třeboň, Czech Republic; 3 Pharmacognosy, Institute of Pharmacy, University of Innsbruck, Innrain 80-82, 6020 Innsbruck, Austria; 4 Department of Ecology and Evolutionary Biology, University of Connecticut, Storrs, CT 06269-3043 USA; 5 Functional Plant Biology, Institute of Botany, University of Innsbruck, Sternwartestr. 15, 6020 Innsbruck, Austria

## Abstract

Ultraviolet (UV) radiation has become an important stress factor in polar regions due to anthropogenically induced ozone depletion. Although extensive research has been conducted on adaptations of polar organisms to this stress factor, few studies have focused on semi-terrestrial algae so far, in spite of their apparent vulnerability. This study investigates the effect of UV on two semi-terrestrial arctic strains (B, G) and one Antarctic strain (E) of the green alga *Zygnema*, isolated from Arctic and Antarctic habitats. Isolates of *Zygnema* were exposed to experimentally enhanced UV A and B (predominant UV A) to photosynthetic active radiation (PAR) ratio. The pigment content, photosynthetic performance and ultrastructure were studied by means of high-performance liquid chromatography (HPLC), chlorophyll *a* fluorescence and transmission electron microscopy (TEM). In addition, phylogenetic relationships of the investigated strains were characterised using *rbc*L sequences, which determined that the Antarctic isolate (E) and one of the Arctic isolates (B) were closely related, while G is a distinct lineage. The production of protective phenolic compounds was confirmed in all of the tested strains by HPLC analysis for both controls and UV-exposed samples. Moreover, in strain E, the content of phenolics increased significantly (*p* = 0.001) after UV treatment. Simultaneously, the maximum quantum yield of photosystem II photochemistry significantly decreased in UV-exposed strains E and G (*p* < 0.001), showing a clear stress response. The phenolics were most probably stored at the cell periphery in vacuoles and cytoplasmic bodies that appear as electron-dense particles when observed by TEM after high-pressure freeze fixation. While two strains reacted moderately on UV exposure in their ultrastructure, in strain G, damage was found in chloroplasts and mitochondria. Plastidal pigments and xanthophyll cycle pigments were investigated by HPLC analysis; UV A- and UV B-exposed samples had a higher deepoxidation state as controls, particularly evident in strain B. The results indicate that phenolics are involved in UV protection of *Zygnema* and also revealed different responses to UV stress across the three strains, suggesting that other protection mechanisms may be involved in these organisms.

## Introduction

Polar regions are characterised by extreme climatic conditions. Organisms living there have to possess adaptations that enable them to survive in such a harsh environment. Numerous abiotic stress factors have been connected with polar climate, including low temperature, drought, nutrient limitation and periodic freeze–thaw cycles during the summer [74]. Among those extreme abiotic factors, solar ultraviolet (UV) radiation seems to be not usually considered a major stress factor in polar regions [23, 24]. Solar elevation decreases towards higher latitudes, and therefore, irradiation is lower in polar regions than in temperate and tropical zones. Moreover, solar rays travel a longer path through the atmosphere in getting to the poles, resulting in a greater proportion of shortwave radiation being absorbed and scattered [23]. Over the last few decades and in light of anthropogenically induced ozone depletion, there has been greater interest in the biological effects of UV radiation on polar organisms [34, 49, 75]. It has been hypothesised that certain polar organisms may be unable to adapt to an increasing UV environment. Moreover, predictions for future scenarios allow speculations about an increase of UV radiation reaching the Earth’s surface particularly in polar regions. These effects are expected to be further enhanced due to climate change [10].

Many damaging effects caused by UV irradiation have been described, of which the dominant targets are DNA and the photosynthetic apparatus, and secondary effects are caused by the production of reactive oxygen species (ROS) [e.g. 14, 19, 62–67, 76]. Photoautotrophic organisms may be especially threatened by increases in UV radiation because solar radiation is essential for their growth and survival. In polar regions, eukaryotic algae are significant primary producers with the ability to inhabit and even dominate practically all habitats [17]. Recently, extensive research has been performed on the UV resistance of polar algae, especially all marine macroalgae [e.g. 27, 30, 37, 38, 52, 53, 61], and the findings have been reviewed by Hanelt et al. [24], Holzinger and Lütz [29] and Karsten et al. [39]. In general, the resistance to UV stress in macroalgae corresponds to their depth zonation [7].

Few studies have examined the adaptation of polar freshwater or semi-terrestrial algae to UV radiation. This is rather surprising because, in habitats such as shallow pools, these algae are subject to relatively high levels of irradiation, low levels of dissolved organic carbon and low temperatures that slow down repair mechanisms [26]. Apart from that, algae from shallow aquatic localities or semi-terrestrial sites can be additionally subject to desiccation [18, 32, 38]. Additionally, in a study of the arctic soil alga *Tetracystis*, extraplastidal carotenoids were found, which shield against irradiation [58]. In another study, Holzinger et al. [31] investigated field-collected samples of the filamentous Zygnematophyceae, *Zygnema* sp. from Svalbard (High Arctic). They concluded that this alga was well adapted to ambient conditions of high irradiation and insensitive to experimentally enhanced UV. Similarly, marine intertidal species of the Trebouxiophyceae, *Prasiola crispa* [28], and the Ulvophyceae, *Ulva* sp. [8] and *Urospora penicilliformis* [61], which need to survive temporarily under almost terrestrial conditions, were also markedly resistant to UV.

Eukaryotic algae possess a range of mechanisms to reduce the impact of UV radiation. In addition to repair mechanisms, protection including UV-screening mechanisms plays an important role. The ability to produce UV-absorbing compounds has two main advantages: First, algae are not limited to the habitats where beneficial physical conditions are available, e.g. dissolved organic carbon or Fe^3+^ ions, which are able to effectively screen UV [4, 70]. Second, they are not impaired by nonspecific attenuation of both UV and visible light [14]. Various substances produced by algae with the ability to screen UV radiation are known, for example, mycosporine-like amino acids (MAAs) [13], secondary carotenoids [42], phenolics [60, 66] or sporopollenin [78].

The goal of the present study was to describe the effects caused by an enhanced UV to photosynthetic active radiation (PAR) ratio on three selected strains of *Zygnema* isolated from polar regions. Algae in the genus *Zygnema* are ideal for such investigations because they grow typically in shallow pools, streamlets or on the surface of wet soils. Moreover, they occur worldwide, including both the Arctic and Antarctica. Even in polar regions, *Zygnema* is quite common and easy to recognise due to its mat-forming growth [25, 31, 40]. As mentioned, no harmful effect of experimental UV exposure on *Zygnema* was observed in previous studies [20, 31], yet, the nature of UV protection in *Zygnema* remains unknown. We expect that the alga obtains UV resistance by the accumulation of phenolic compounds, which can be analysed by high-performance liquid chromatography (HPLC). The UV-screening ability of phenolic substances found in some members of the class Zygnematophyceae has already been discussed [18, 60]. Moreover, high amounts of water-soluble pigments, probably also phenolics, have been reported to occur in *Zygnema cruciatum* [22], but their role in UV screening has not been tested. In this study, we examined if the production of phenolics in *Zygnema* is enhanced by experimental UV exposure. In addition, we determined pigment contents of chlorophylls and xanthophyll cycle pigment and measured photosystem (PS) II efficiency, and we also observed the ultrastructure of the cells to prove the effectiveness of UV tolerance. Finally, we established the phylogenetic relationships of the three strains of *Zygnema* according the *rbc*L sequences.

## Material and Methods

### Origin of the Strains

Three strains of *Zygnema*, originating from the polar regions, were chosen for our experiments. Two of the strains, strain B (CCALA 976) and strain G (CCALA 977), were isolated in 2010 on Svalbard (High Arctic) from shallow seepage pools in the Petunia Bay (78°40′ N, 16°30′ E) and deposited in the Culture Collection of Autotrophic Organisms in Třeboň, Czech Republic (CCALA, www.butbn.cas.cz/ccala/index.php). Strain E (CCCryo 278-06) was obtained from the CCCryo culture collection in Potsdam-Golm (cccryo.fraunhofer.de) isolated in 2006 from a meltwater pool north of Artigas Base freshwater lake (also known as Lago Uruguay, Lake Profound or Artigas Base freshwater lake), Fildes Peninsula, Maxwell Bay, King George Island, South Shetland Islands, Antarctica. Prior to the experiment, all strains were cultivated in liquid Bold’s basal medium (BBM) [9] at 15 °C with continuous light regime (∼38 μmol m^−2^ s^−1^).

### DNA Sequencing and Phylogenetic Analysis

DNA was isolated from the three polar strains of *Zygnema* (B, E and G) using the PowerPlant DNA Isolation Kit (MO BIO Laboratories, Inc., Carlsbad, CA, USA) bacically as described in [36]. Polymerase chain reaction (PCR) amplification of the *rbc*L gene was performed using primers M28F, M1161R or M1390R [43, 47] or with newly designed primers 443F 5′-TCCAAGGTCCTCCTCATGGTATCC-3′ and 1263R 5′-ACGGTTTGCACCTGCTCCAGGT-3′. PCR conditions were as listed in [35]. Products of cycle sequencing were analysed on an ABI 3100 DNA Sequencer™ (Applied Biosystems, Foster City, CA, USA), with individual reads compiled into contigs in Sequencher 4.5 (Gene Codes Inc., Ann Arbor, MI, USA) and edited manually to resolve any ambiguity. *rbc*L sequences from the study strains of *Zygnema* were compared to the NCBI database through BLAST searches [3], and the resulting top three matches of each new *Zygnema* sequence were used to produce an alignment. Published *rbc*L sequences from two members of Desmidiales, *Cylindrocystis* and *Mesotaenium*, were used as outgroup. The *rbc*L alignment includes 1,384 nucleotide positions, with a total of 146 parsimony informative sites and 89 parsimony sites for the in-group taxa only. Maximum likelihood (ML) analysis and the ML bootstrap analysis (200 replicates) were performed in PAUP* [72]. The Bayesian analysis was done in Mr. Bayes [33]. The model of sequence evolution used in the ML and Bayesian analyses was selected by jModelTest v0.1 [54] under the Akaike Information Criterion as GTR + I + gamma. Two independent Bayesian analyses each were run for 1.1 × 10^6^ generations with one cold plus three heated chains, with a subsample frequency of 200. Convergence within and between runs was determined using Tracer v1.4.1 [56]. Trees from the initial 10^5^ generations were discarded as burn-in before determining the majority-rule consensus tree.

### Experimental Cultivation

During the experiments, the algae were exposed on agar-solidified BBM [9] plates, three parallel Petri dishes for control and three for UV-exposed samples for each strain. Regularly, a thin layer of liquid medium was added to prevent additional desiccation stress. The dishes were placed into a climate chamber (Percival PGC_6L, USA), with temperature set to 15 °C and a continuous illumination of 38.1 ± 3.9 μmol m^−2^ s^−1^ (PAR) on average. Subsequently, UV was induced by using a combination of a UV A (Sylvania BL 350, Havells Sylvania, UK; with UV A in the range of 315–400 nm with a peak at 352 nm; for spectrum see the manufacturers web site: www.spezilamp.de/sylvania-mini-lynx-longlife-p-8556.html) and a UV B (LX-363 Drago-lux 12.0, Germany; www.dragonterraristik.de/Technik/Licht-Waerme-UV/DRAGO-LUX-DELUXE-COMPACT-120-36-W-24::1793.html) fluorescence tube. Acrylic glass filters (Plexiglas® XT) with a cut-off wavelength of approximately 280 nm were placed over the UV exposure and an additional filter with a cut-off at approximately 400 nm over the control samples, respectively. The amount of incident light energy slightly varied within the whole experimental area, that is why the dishes were shifted in a circular manner twice a day to provide overall identical conditions for all replicates. The aim of the exposure was to study the effects caused by enhanced UV/PAR ratio, but not to simulate the spectral proportions of outdoor solar irradiation. Thus, the mean intensities of UV irradiation were 3.22 ± 1.69 W m^−2^ UV A and 0.018 ± 0.015 W m^−2^ UV B measured with a PMA2100 (Solar Light, USA). This gives a ratio of UV B:UV A of approximately 1:160, resulting in a predominant UV A treatment. The UV irradiation was provided for 8 h day^−1^. The UV penetrating the cut-off filter was negligible (0.28 ± 0.25 W m^−2^ UV A and 0.0003 ± 0.0002 W m^−2^ UV B).

### Light and Transmission Electron Microscopy

Potential cellular changes induced by UV stress were observed on the morphological and ultrastructural level. A Zeiss Axiovert 200M Light Microscope (Carl Zeiss AG, Oberkochen, Germany) equipped with a Zeiss Axiocam MRc5 camera was used for capturing micrographs.

Control and 4-day UV-exposed samples were high-pressure frozen and freeze-substituted according to the methods of Aichinger and Lütz-Meindl [2] with modifications. Prior to freezing, samples were transferred to 150 mM sucrose for 1 min. Freeze substitution was carried out in 2 % OsO_4_, 0.05 % uranyl acetate in acetone at −80 °C for 60 h. Temperature was raised to −30 °C at a d*T* of 10 °C h^−1^ within 5 h and substitution continued for 4 h at −30 °C. Subsequently, temperature was raised to 20 °C at a d*T* of 2.5 °C h^−1^ for 20 h, and samples were kept at 20 °C for 10 h. Samples were then transferred to propylene oxide and embedded in low-viscosity embedding resin (Agar Scientific, England). For transmission electron microscopy (TEM), ultrathin sections (∼60 nm) were prepared with a Leica Ultracut, counterstained with uranyl acetate and Reynold’s lead citrate and investigated at a Zeiss LIBRA 120 transmission electron microscope at 80 kV. Images were captured with a ProScan 2k SSCCD camera.

### Photosystem II Efficiency

The physiological state of the photosynthetic apparatus was determined by measuring the maximum quantum yield of PS II photochemistry in a dark-acclimated state (*F*_V_/*F*_M_). The samples were taken immediately after the end of the experiment (i.e. after 7 days of exposure to UV/PAR) in five replicates within each of the three Petri dishes (*n* = 15) per strain, dark-adapted for 30 min and then *F*_V_/*F*_M_ was measured with a Handy PEA (Hansatech Instruments, UK).

### High-Performance Liquid Chromatography

Control samples and samples exposed for 7 days to the previously mentioned UV regime were investigated by HPLC. At the end of the experiment, the biomass from individual Petri dishes was filtered onto glass fibre filters (Whatman GF/C), freeze-dried for 48 h and the dry weight measured. When sufficient biomass could be obtained (strains E and G), two filters were made from each Petri dish (*n* = 6). Only one filter per Petri dish was made for strain B (*n* = 3).

Chlorophylls and carotenoids were quantified by HPLC according to Remias et al. [57]. Briefly, freeze-dried filters were ground with a Micro-Dismembrator (Sartorius) in pre-cooled Teflon jars with a quartz ball and extracted in *N*,*N*-dimethylformamide. After centrifugation and prior to injection, 50 % methanol was added to the extract (1:2). The HPLC system (Agilent ChemStation 1100) was run with a binary solvent gradient and using a LiChroSpher column (RP-C18, 150 × 4 mm). The detection wavelength was 440 nm. Peak assignment was carried out with individual retention times and absorption spectra in comparison to available pigment standards (DHI C14 Centralen, Denmark).

The samples were also screened for polar, soluble phenolics by alternative extraction of the ground cells with methyl *tert*-butyl ether, followed by phase separation with 20 % methanol. The latter phase was prepared for HPLC injection by centrifugation (15,000×*g*, 10 min) and filtration through 0.45 μm regenerated cellulose syringe filters. Analysis was performed with the same HPLC system as for pigments, however, using a Phenomenex Synergi Hydro column (RP-C18, 150 × 2 mm) with oven set at 30 °C, pump 0.3 ml min^−1^, solvent A: water + 0.5 % formic acid, solvent B: methanol + 0.5 % formic acid. The binary linear gradient was as follows: start 0 % B, 40 min 100 % B and then 8 min post-run with 100 % A. The injection amount was 20 to 40 μl; typical peaks had retention times between 5 and 25 min. A detection wavelength of 280 nm was used for semiquantitative peak integration to gain the relative phenol content per sample. The amount of phenolics was calculated as the total peak areas normalised to the algal dry weight.

### Data Analyses

Data were analysed using the general linear model (GLM) analysis of variance (ANOVA) with nested design because of the hierarchical structure of the design. The effects of factors ‘strain’ (B, E and G) and ‘UV treatment’ (UV and control) were tested. For each factor combination, three Petri dishes were used. Five replicate measurements of *F*_V_/*F*_M_ were done in each dish and one to two filters were prepared from each dish for the HPLC analysis. Therefore, the effect of individual Petri dishes was nested in ‘strain’. ‘Petri dish’ was considered as a random effect factor and both ‘strain’ and ‘UV treatment’ were considered as factors with fixed effects. When significance of an effect was proved by GLM ANOVA, additional Tukey’s test was performed for post hoc pairwise comparisons. All statistical analyses were performed in Statistica for Windows, Version 10, Statsoft.

## Results

### Molecular Characterisation

**Figure 1:**
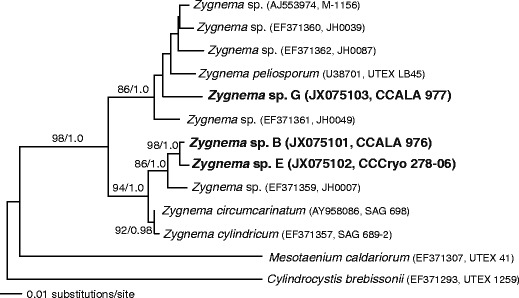
ML tree (score −lnL = 4,104.333) from phylogenetic analysis of *rbc*L sequence data from different strains of *Zygnema* plus two related saccoderm desmid genera. Taxon labels include GenBank accession numbers and strain designations. *Labels listed in boldface font* were obtained for this study. The GTR + gamma model parameter values were set during the search: RA-C = 3.0900652; RA-G = 7.7050449; RA-T = 7.7120372; RC-G = 2.7439213; RC-T = 28.160416; RG-T = 1; gamma shape = 0.813032; pinvar = 0.441081. ML bootstrap support values (out of 200 replicates) are indicated, followed by Bayesian posterior probabilities. Scale bar corresponds to the number of expected substitutions/site

The *rbc*L sequences of the three new *Zygnema* strains were deposited in GenBank under accession numbers JX075101 (strain B), JX075102 (strain E) and JX075103 (strain G). The sequences obtained were 721 (strain G), 1,302 (strain B) and 1,358 (strain E) nucleotides in length. Phylogenetic analysis of the new *rbc*L data with related published *rbc*L sequences (Fig. 1) demonstrated that strains B and E are closely related (they differ by seven nucleotides, representing 0.5 % difference overall) and together are related to a strain of *Zygnema* cf. *insigne* isolated by John Hall (JH0007) [71]. The strain *Zygnema* sp. G is in a separate clade from strains B and E, and instead is most closely related to *Zygnema peliosporum* (UTEX LB45). The distinction of strains B and E from G is supported strongly by both ML bootstrap and Bayesian analysis (Fig. 1).

### Light Microscopy

**Figure 2:**
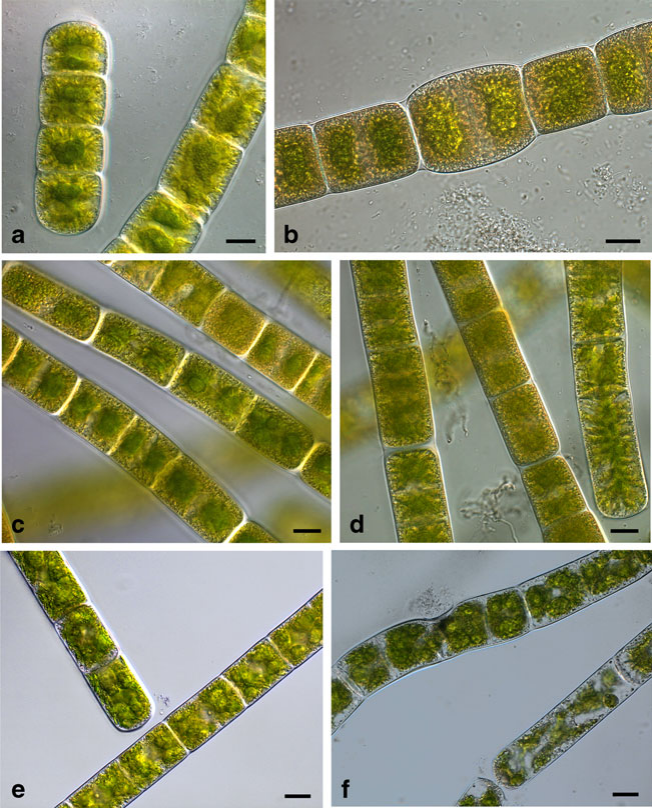
Light microscope images of the investigated *Zygnema* strains. **a** strain B—control, **b** strain B—after UV exposure, **c** strain E—control, **d** strain E—UV exposure, **e** strain G—control, **f** strain G—UV exposure. Scale bars, 10 μm

All three investigated strains had typical *Zygnema* appearance with two star-shaped chloroplasts per cell, each containing a pyrenoid in the centre of the chloroplast (Fig. 2a, c). All samples, including PAR-only, had a remarkably dense cytoplasm with chloroplasts practically filling up the whole intracellular space. In strain B, the cells were considerably browner after UV treatment (Fig. 2b), which could be a result of an increased number of vacuoles at the cell periphery. On the other hand, there was no visible difference between control and UV-treated cells in strain E, both cultures appeared similar; UV-exposed samples remained unaltered (Fig. 2c, d). On the contrary, in strain G, obvious damages could be observed after UV exposure (Fig. 2f). Most of the cells lost their cytoplasmic streaming completely and chloroplasts disintegrated into smaller, often globular pieces.

### Transmission Electron Microscopy

**Figure 3:**
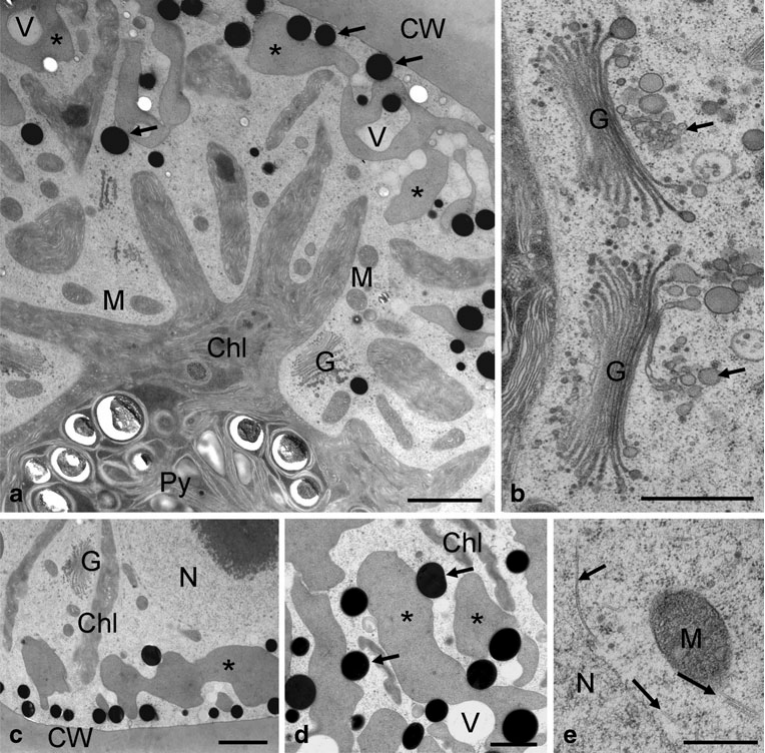
Details of the ultrastructure of control cells of *Zygnema* sp. B. **a** Star-shaped chloroplast in the centre of the cell, medium electron-dense compartments (*asterisks*) and electron-dense particles (*arrows*) at the cell periphery; **b** Golgi bodies in close contact with chloroplast, a TGN is clearly visible (*arrows*); **c** cell periphery with medium electron-dense compartments (*asterisk*), nucleus in the centre of the cell; **d** multiple shapes of medium electron-dense compartments (*asterisks*) and electron-dense particles (*arrows*); **e** microtubules (*arrows*) close to the nucleus and mitochondrion. *Chl* chloroplast, *CW* cell wall, *G* Golgi bodies, *M* mitochondrion, *N* nucleus, *Py* pyrenoid. Scale bars: **a**, **c** 2 μm; **b**, **d** 1 μm; **e** 500 nm

**Figure 4:**
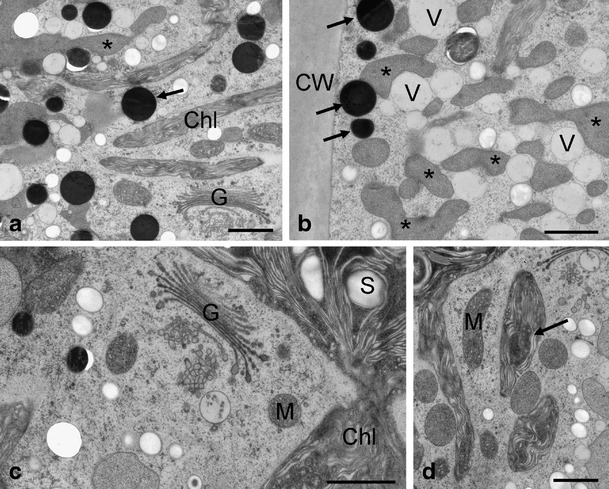
Transmission electron micrographs of *Zygnema* sp. B after UV exposure. **a** Chloroplast lobes, Golgi bodies, medium electron-dense compartments (*asterisk*) and electron-dense particles (*arrow*) appear unchanged; **b** while medium electron-dense compartments (*asterisks*) and electron-dense particles (*arrows*) appear unchanged, additional occurrence of electron-translucent vacuoles was found; **c** Golgi body with normal appearance, chloroplast with starch grain; **d** unchanged mitochondria, chloroplast lobe with electron-dense inclusion (*arrow*). *Chl* chloroplast, *CW* cell wall, *G* Golgi bodies, *M* mitochondria, *S* starch grain *V* vacuole. Scale bars: **a**–d 1 μm

When viewed by TEM, *Zygnema* sp. B showed star-shaped chloroplasts in the centre of the cells (Fig. 3a). The chloroplasts had a central pyrenoid with starch grains and several lobes protruding towards the cell periphery (Fig. 3a). In the cell periphery, medium electron-dense compartments and electron-dense particles were observed (Fig. 3a, c, d) and small vacuoles were found (Fig. 3a, d). Mostly in close vicinity of the chloroplast, Golgi bodies with at least 10 cisternae were visible (Fig. 3b). At the Golgi bodies, a clear distinction between *cis*- and *trans*-side was possible, the latter giving rise to the *trans*-Golgi network (TGN; Fig. 3b). The cell centre contained a nucleus (Fig. 3c) with a marked nucleolus. The vacuoles in the cell periphery were polymorphic (Fig. 3c, d); electron-dense particles had a diameter of approximately 400–600 nm (Fig. 3d). Close to the nucleus, microtubules were occasionally observed; mitochondria showed an internal structure with cristae (Fig. 3e). When *Zygnema* sp. B cells were exposed to UV, only little modifications of the ultrastructure were observed (Fig. 4). The chloroplast lobes covered medium electron-dense compartments, and electron-dense particles were still evident (Fig. 4a, b). However, the amount of vacuolisation increased (Fig. 4b). In general, neither Golgi bodies nor mitochondria were altered (Fig. 4c). The chloroplast still contained starch grains (Fig. 4c); only occasionally electron-dense spots were observed within the chloroplast lobes (Fig. 4d).

**Figure 5:**
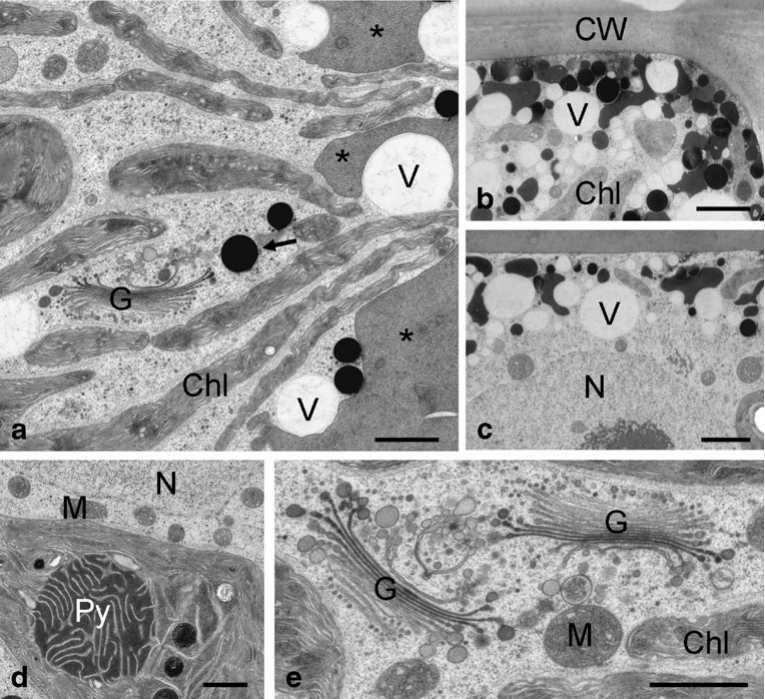
Details of the ultrastructure of control cells of *Zygnema* sp. E. **a** Long and narrow chloroplast lobes covered by large medium electron-dense compartments (*asterisks*) and electron-dense particles (*arrow*); **b** cortical area with electron-dense particles and vacuoles; **c** central area of the cell with nucleus and part of the nucleolus (*arrow*), vacuoles in the cell cortex; **d** chloroplast detail with pyrenoid, mitochondria and nucleus; **e** detail with Golgi bodies, mitochondria and chloroplast lobes. *Chl* chloroplast, *CW* cell wall, *G* Golgi body, *M* mitochondrion, *N* nucleus, *Py* pyrenoid, *V* vacuole. Scale bars: **a**, **d**, **e** 1 μm; **b**, **c** 2 μm

**Figure 6:**
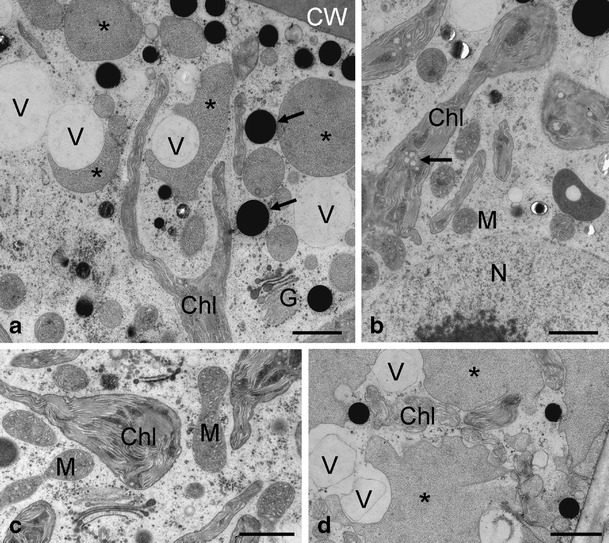
Transmission electron micrographs of *Zygnema* sp. E after UV exposure. **a** Detail of the cell cortex with chloroplast lobes, substantial amount of vacuolisation, medium electron-dense compartments (*asterisks*) and electron dense particles (*arrows*); **b** central area, nucleus with nucleolus, mitochondria intact, the chloroplasts contain electron-dense areas and plastoglobules (*arrow*); **c** mitochondria with normal appearance, chloroplast partly swollen; **d** extensive medium electron-dense compartments (*asterisks*) in the cell cortex, vacuoles and chloroplast lobes are found in the same area. *Chl* chloroplast, *CW* cell wall, *G* Golgi body, *L* lipid body, *M* mitochondrion, *V* vacuole. Scale bars: **a**–**d** 1 μm

*Zygnema* sp. E control cells (Fig. 5) had particularly long and narrow chloroplast lobes, covered by large medium electron-dense compartments. Electron-dense particles and electron-translucent vacuoles were also observed in the cell periphery (Fig. 5a, b). The nucleus showed a distinct nucleolus, which is shown in part in Fig. 5c. The pyrenoid had a characteristic shape, with thylakoid membranes penetrating the central electron-dense area (Fig. 5d). Golgi bodies (Fig. 5e) were similar to those described for *Zygnema* sp. B. Upon exposure of *Zygnema* sp. E to UV radiation, again, no drastic changes of the ultrastructure were observed (Fig. 6). Medium electron-dense compartments, vacuoles and electron-dense particles remained in the cell periphery. Golgi bodies and mitochondria remained intact (Fig. 6a). Only occasionally, chloroplasts showed accumulations of plastoglobules or electron-dense areas (Fig. 6b, c). The nucleus remained visibly intact with one nucleolus (Fig. 6b). In some cases, particularly large vacuoles were observed (Fig. 6d).

**Figure 7:**
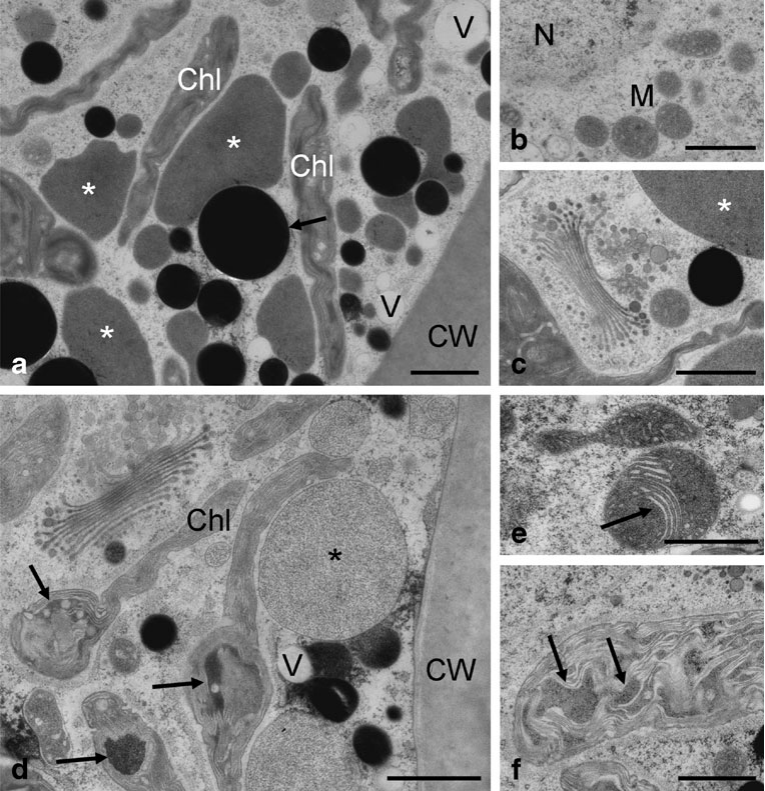
Details of the ultrastructure of *Zygnema* sp. G control cells (**a**–**c**) and cells after UV exposure (**d**–**f**). **a** Chloroplast lobes between medium electron-dense compartments (*asterisks*), vacuoles and electron-dense particles (*arrow*) in the vicinity of the cell wall; **b** central area with nucleus and mitochondria; **c** Golgi body next to a chloroplast lobe and medium electron-dense compartment (*asterisk*); **d** cortical area with altered chloroplast with swellings (*arrows*), partially filled with electron-dense content or plastoglobules, medium electron-dense compartment (*asterisk*); **e** altered mitochondria with rearranged parallel-oriented cristae (*arrow*); **f** chloroplast with electron-dense contents (*arrows*). *Chl* chloroplast, *CW* cell wall, *G* Golgi body, *M* mitochondrion, *N* nucleus. Scale bars: **a**–**f** 1 μm

While *Zygnema* sp. G control cells (Fig. 7a–c) had a similar appearance as the previously mentioned control cells, the exposure to UV lead to more drastic consequences in this strain (Fig. 7d–f). Control cells contained narrow chloroplast lobes between medium electron-dense compartments, electron-dense particles and vacuoles (Fig. 7a). Nucleus, mitochondria and Golgi bodies had the expected appearance in TEM control samples (Fig. 7b, c). *Zygnema* sp. G exposed to UV showed swellings of the chloroplasts with plastoglobules and partially electron-dense areas (Fig. 7d, f); mitochondria were particularly electron-dense and showed a rearrangement of cristae (Fig. 7e).

### Photosystem II Efficiency

**Figure 8:**
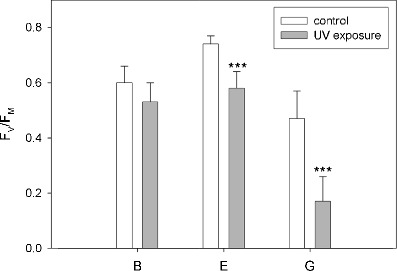
Maximum quantum yield of PS II in dark-adapted state for both control and UV-exposed samples; mean values + SD. Significant differences (*p* < 0.001) between control and UV-exposed samples were found in strains E and G and are marked with *three asterisks* (*n* = 15)

The mean values of the maximum quantum yield of PS II (*F*_V_/*F*_M_) were substantially lower after UV exposure than in the control (Fig. 8). However, the response to UV differed among the tested strains, and this decrease was significant only in strain E and strain G. The results of the statistical analyses testing the difference of measured values of *F*_V_/*F*_M_ are presented in Table 1; the pairwise comparisons confirmed the significant difference between control and exposure in strain E (*p* < 0.001) and strain G (*p* < 0.001), but not in strain B (*p* = 0.086). For comparison of the values in individual strains, see Fig. 8.

**Table 1:** Summary of the results of statistical analyses (GLM, *p* values of nested ANOVA)

Effect	*df*	*F*_V_/*F*_M_	Total phenolics	Chlorophylls *a* + *b*	V + A + Z	Other primary carotenoids	Deepoxidation state
UV treatment	1	<0.0001	0.0012	0.9884	0.0813	0.2829	0.0473
Strain	2	0.0001	0.0037	0.0816	0.0058	0.0129	0.0022
Petri dish (strain)	6	0.0319	0.5595	0.15	0.3896	0.2545	0.3961
Strain × treatment	2	<0.0001	0.0404	0.9163	0.7539	0.3247	0.0776

‘UV treatment’ and ‘strain’ were considered as fixed effects, ‘Petri dish’ was considered as a random effect

*Petri dish (strain)* Petri dish nested in strain, *F*_*V*_ */F*_*M*_ PS II efficiency, *V + A + Z* xanthophyll cycle pigments, *Deepoxidation state* the ratio (A + Z)/(V + A + Z)

Interestingly, *F*_V_/*F*_M_ differed significantly among individual controls as well, showing a considerable level of stress response even without experimental UV treatment—most pronounced in strain G. Apparently, this strain was already impaired under control conditions. Low values (around 0.2) measured after UV exposure indicated that the photosystems were practically destructed.

### Plastidal Pigments

The content of plastid-bound pigments was measured by HPLC. The pigments were categorised into three groups: First, chlorophyll *a* and *b*, including some degenerative phaeophytin derivates, which occurred in several samples; second, xanthophyll cycle pigments, i.e. violaxanthin (V), antheraxanthin (A) and zeaxanthin (Z); and third, the remaining primary carotenoids, including beta-carotene, lutein and neoxanthin. Finally, the deepoxidation state of xanthophyll cycle (A + Z)/(V + A + Z) was estimated.

**Figure 9:**
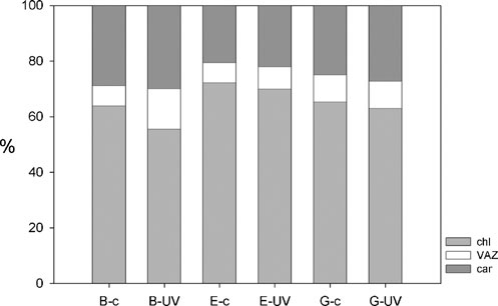
Proportion of individual pigment groups to the total chloroplast pigment content for each strain and treatment (mean values). *Chl* chlorophylls *a* and *b*, *VAZ* violaxanthin, antheraxanthin and zeaxanthin (xanthophyll cycle pigments), *car* other primary carotenoids

Neither of the investigated group of pigments differed significantly between controls and UV-exposed samples (Table 1). However, the total pigment content per dry weight was significantly lower in strain B than in the other two strains (Table 2; *p* < 0.005). Therefore, the relative pigment compositions for each individual strain are presented (Fig. 9).

**Table 2:** Total pigment content of the investigated strains of *Zygnema* (in micrograms per gram dry weight) and deepoxidation state (A + Z)/(V + A + Z)

Strain	B		E		G	
Treatment	Control	UV	Control	UV	Control	UV
Total pigment—mean	0.8076	0.8750	2.8083	2.7018	2.5265	3.0529
Total pigment—SD	0.3834	0.1060	0.7915	1.1058	1.3739	0.6734
Deepoxidation state—mean	0.2947	0.5926	0.4320	0.4615	0.7814	0.7707
Deepoxidation state—SD	0.1139	0.1058	0.0710	0.0700	0.0253	0.2015

Mean values are given as well as standard deviations

When viewed from the deepoxidation state of the cultures, significant differences were revealed between UV-treated samples and controls (Table 1), showing that the UV samples were in higher deepoxidation state than the controls. The individual strains differed as well, as strain G was significantly more deepoxidised than the other two (Tables 1 and 2). Finally, virtually no secondary carotenoids were detected by using the same HPLC protocol as for pigment analysis.

### Phenolic Compounds

By HPLC, all three *Zygnema* strains showed the presence of several compounds with the ability to screen UV radiation. The putative phenolic nature of these peaks was derived from their characteristic spectral absorption maxima around 280 nm typical for aromatic compounds and also from their water-soluble, hydrophilic nature.

**Figure 10:**
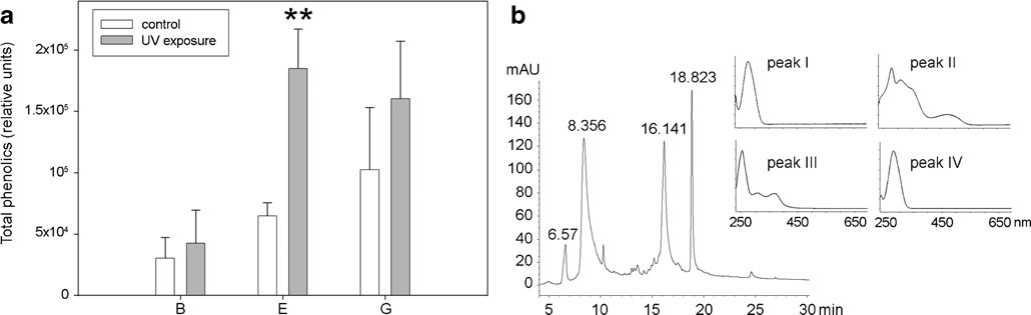
**a** Total content of phenolic compounds expressed as peak area per 1 mg dry weight. Strain E control and 7-day UV-exposed samples differ significantly (*p* = 0.001) in their amount of phenolics, as indicated with *two asterisks*; the differences in the other strains are not significant (*n* = 6 for E and G, *n* = 3 for B). Mean values + SD. **b** Representative HPLC chromatogram at 280 nm of a UV-exposed culture (*E*) revealing several different phenolic peaks. The control samples contained these compounds as well, only in lower concentrations per dry weight. Online absorption spectra of the four largest peaks are also given

The mean values of the content of phenolic compounds were always higher in UV-exposed samples than in the controls (Fig. 10a), but the effect of UV was different among individual strains. The detailed results of statistical tests can be seen in Table 1. Markedly, the pairwise comparisons clearly showed the difference between control and exposure to be significant within only one of the strains tested—strain E (*p* = 0.001). In general, strain B possessed fewer phenolics per dry weight than the other two strains. Furthermore, the same phenolics were present also in control samples, suggesting that certain amounts of protective compounds are present independently of environmental UV stimuli. Figure 10a illustrates the summarised peak areas of the phenolics for each species and their increase after UV exposure.

Figure 10b demonstrates a representative HPLC chromatogram with the phenolic peaks of *Zygnema* strain E after exposure. The first and the last main peaks (retention times, 6.57 and 18.823 min) have HPLC–online absorption maxima of approximately 278 nm, thus absorbing mainly UV B radiation. Two further main peaks (8.356 and 16.141 min) show an additional absorption in the UV A region (shoulder at 352 nm and a minor maximum at 367 nm, respectively). The second peak (retention time 8.356) can be even seen in the visible (VIS) light region with a minor maximum at 473 nm. This fact corresponds most likely with the yellowish colour of the aqueous extracts. All the minor peaks had similar absorption spectra. The control samples contained all these compounds as well, however, in lower concentrations per dry weight. The other two species had very similar chromatograms (data not shown).

## Discussion

We investigated the effect of UV on the cell structure and ultrastructure, the physiological performance and the production of intracellular soluble compounds in three polar strains of *Zygnema*. All strains were isolated from polar habitats and, therefore, a comparable level of stress resistance could be speculated about. After UV irradiation, we found a significant increase in accumulation of phenolic compounds in strain E and a significant decrease in PS II efficiency in strains E and G, when compared to control cells. Both effects are stress responses, however, occurring to a different extent in the investigated strains.

The UV scenario created in our experiments did not reflect the natural solar spectrum, but included a shift in the ratio of UV to PAR, a method that is widely used for provoking UV effects in algae [e.g. 28, 31, 38, 52, 61, 63, 79]. Despite the experimental limitations, we found significant effects of UV treatment on the phenolic compounds in strain E. To our knowledge, this is the first study to use an HPLC protocol rather than spectrophotometric assays to quantify changes of phenolic compounds in streptophyte green algae after exposure to UV; recently, a red algal species, *Hypnea*, was investigated concerning phenolic compounds by HPLC [66].

### Phylogenetic Relationships

One possible explanation for the differences observed among the investigated strains is their phylogenetic relationship. The species concept of the genus *Zygnema*, and in nearly all algae, is dominantly based on morphological characteristics [35], but it has been shown that traditional classification may not be consistent with phylogeny [71]. Further, the morphological distinction among *Zygnema* species rests on information from zygospores. Sexual reproduction was not observed in our strains and it was noted that other *Zygnema* from polar regions do not reproduce sexually [31, 40].

Therefore, the three strains under investigation were characterised by phylogenetic analysis of their *rbc*L sequences. The genus *Zygnema* contains over 137 species. The systematics of only a small number of *Zygnema* species was recently clarified using analysis of *rbc*L and cox3 data, but there are many species for which molecular data are not available [71]. Thus, we were unable to assign the strains used in this study to species. That said, the published sequences generated by this study should ultimately allow taxonomic assignment of the strains in the future and the information provided by these polar strains contributes to the understanding of the diversity and biology of the genus *Zygnema*. Interestingly, the two Arctic strains, *Zygnema* sp. B and G, are not most closely related. Instead, *Zygnema* sp. B is very closely related to the Antarctic strain (E) and to *Zygnema* cf. *insigne*.

### Structural and Ultrastructural Aspects

Morphologically, samples of the three strains under control conditions looked similar. They all had a markedly condensed protoplasmic content and contained quite a high amount of small coloured bodies. Such a cytosolic appearance is not typical for young vegetative cells of *Zygnema* and resembles so-called pre-akinetes [46]. Many algae produce such modified vegetative cells by accumulating lipids and other metabolic products and by thickening the cell walls. Akinetes are formed at the onset of unfavourable conditions, such as nutrient starvation, and are considered resistant resting stages [15]. Markedly, natural populations of *Zygnema* sp. containing a high amount of lipid bodies have already been reported from the Arctic and Antarctica [25, 31]. Also, the Zygnematophycean freshwater ice alga *Mesotaenium berggrenii* that survives harsh environmental conditions at glacier surfaces without making cysts contains high amounts of purpurogallin-derived, phenolic compounds [60]. It seems that such a cytology is an adaptation to extreme climatic conditions, where cells could be suddenly exposed to stress scenarios.

To date, a limited number of studies have addressed UV effects on the ultrastructure of freshwater algae from polar habitats [28, 31, 61], whereas red and brown algae are better studied (for a summary, see Karsten et al. [39]). The desmid *Micrasterias denticulata* was remarkably resistant to UV B; alterations in the ultrastructure were seen only as an effect of irradiation with wavelengths lower than 284 nm [44, 48].

The ultrastructural changes found in the present study as a consequence of UV exposure were moderate in strains B and E, but pronounced in strain G, where serious damage could be observed, especially in chloroplasts and mitochondria. Damage to these organelles is usually the first sign of UV-induced damage in sensitive species [24, 29]. While an earlier study on arctic *Zygnema* was made with field-collected samples followed by an exposure to an increased UV to PAR ratio only for 24 h [31], the present study employed cultivated samples exposed for 4 days to UV (8 h day^−1^) irradiation. Moreover, all earlier studies used conventional chemical fixation [28, 31, 61], but with the laboratory-grown samples, we were able to use sophisticated high-pressure freezing, followed by freeze substitution, a method that is generally accepted to allow better preservation of the ultrastructure.

We speculate that the electron-dense particles with a diameter of 400–600 nm frequently found in the cell cortex contain phenolics. These structures have a similar appearance like physodes, phlorotannin/phenolics containing structures frequently found in brown algae [26, 68, 69]. In the present study, most of these electron-dense particles appeared round, likely due to better preservation by high-pressure freezing. In an earlier study, similar electron-dense structures with irregular outer shape, likely caused by shrinkage processes during dehydration, were detected in arctic *Zygnema* after chemical fixation [31]. We, therefore, assume that these substances are not lipids, as one could conclude by their electron density. Moreover, electron-dense structures have been described by McLean and Pessoney [46] as ‘inclusions’ in beginning akinetes of older *Zygnema* sp. cultures. These structures were evident in untreated as well as in UV-treated cells in our study, which goes along with the finding that phenolics were also detected in both by HPLC analysis, even after prolonged UV exposure. Moreover, the irregularly shaped medium electron-dense compartments in the cell cortex could contain phenolic compounds as well due to the hydrophilic nature of phenolics. However, we could not observe a quantitative enhancement of these medium electron-dense compartments in UV-exposed cells. The nature of these compartments remains obscure; they might either be some sort of lytic vacuoles, which could only be proven by investigating their contents. Due to the texture of these medium electron-dense compartments, one could even speculate that they are thylakoid-free parts of the chloroplast; however, we did not observe any connection of these compartments with the chloroplast.

There was little evidence for the accumulation of lipids based on TEM, which agrees with the observations of Bakker and Lokhorst [5], who also did not show accumulation of lipids in cultured *Zygnema* cells. This is in contrast to field-grown cells, which contain massive amounts of medium electron-dense bodies (‘grey lipid bodies’), likely induced by nutrient starvation or harsh environmental conditions of the Arctic [31].

### Physiological Effects of UV Irradiation

The presence of putative phenolic compounds was proven in all three *Zygnema* strains by using HPLC in control samples, as well as in 7-day UV-exposed samples; the prolonged exposure time in comparison to the TEM investigations had methodical reasons, but we speculate that this prolonged time caused only a quantitative increase in the amount of phenolics. This is in contrast to earlier reports, where only spectrophotometric assays (e.g. Folin–Ciocalteu) were used for a more imprecise determination [e.g. 18, 20, 41]. The presence of such phenolics, which are not common in freshwater microalgae, supports the hypothesis that their production might be crucial in UV protection of *Zygnema*. In all three strains, the mean value of total phenolic content rose after UV exposure. Nevertheless, only in strain E was this increase significant. The lack of significance in *Zygnema* sp. B and G was mainly due to large variation in phenolic content among individual replicates. As a matter of fact, in strain B, not enough biomass was available for more replications.

The screening effects of phenolics are enhanced by the fact that they are stored in electron-dense particles and vacuoles at the cell periphery, therefore protecting presumably chloroplasts and other organelles in the centre of the cells. This arrangement of organelles was confirmed in our *Zygnema* strains by TEM. High accumulation of secondary pigments in cytoplasmic compartments to shade the chloroplast was observed in other algae, too [29, 59]. Moreover, some of the phenolic compounds revealed in *Zygnema* also have an absorption in the VIS region, causing a yellowish to brownish colour of the vacuoles and of the aqueous extracts, respectively. Consequently, such compounds may also serve as protectants against excessive VIS irradiation, which otherwise could cause photoinhibition or intracellular ROS production. Stancheva et al. [71] found that zygotes of *Zygnema* exhibit a yellow, brownish or bluish secondary colouration, probably caused by such phenolic pigments. Strain E, which had the highest levels of phenolics after UV exposure, reached the highest values of *F*_V_/*F*_M_, suggesting a protective role of the phenolics for photosynthetic apparatus. Accordingly, the best photosynthetic rate (rETR) and *F*_V_/*F*_M_ values were found in cells of the chlorophyte *Zygnemopsis decussata* with high content of phenolics [18].

Remarkably, the strains investigated in this study also produced phenolic compounds without UV exposure; the values were highest in strain G, which could point towards influence by other stress. This indicates an accumulation in advance of any harmful irradiation events, probably similarly as in *Zygogonium ericetorum*, which exhibits pinkish vacuoles [32]. Phenolics are widely distributed in brown algae as phlorotannins [1, 16, 50]. In non-Streptophycean green algae, phenolics are not widespread, only few examples like the marine green alga *Dasycladus vermicularis* are known to contain phenolics [51]. In contrast, these compounds have been found in members of the Zygnematophyceae [12, 60], which might be explained by their close relationship to land plants [6, 77]. In addition, a high amount of uncharacterised pigments was detected when attempting to isolate RNA from *Z. cruciatum* [22]. It could be speculated that these pigments were also phenolics, but there are many other compounds in the aqueous phase. Passive UV absorption is a nature of phenolic substances, given by their structure containing aromatic groups, but their original function in regards of plant physiology may be different [14]. Thus, the accumulation of phenolics is triggered not only by UV irradiation [50] but by different environmental factors (salinity, PAR and temperature) as well [51]. In fact, the applied temperature of 15 °C during the experiments could contribute to higher repair rates under these relatively low UV intensities.

In this study, a significant (*p* < 0.001) decrease of PS II efficiency was observed after UV exposure in strains E and G, but not in strain B. This parameter is widely used as an indicator of quantum efficiency of PS II photochemistry and gives a good measure of photoinhibitory damage caused by environmental stresses [11, 45]. On the contrary, in the study of Holzinger et al. [31], no photoinhibition was observed at 926 μmol photons m^−2^ s^−1^ in field-collected samples of *Zygnema* from Svalbard. Also, Germ et al. [20] showed no decrease in *F*_V_/*F*_M_ under UV B exposure. In other species, however, it has already been shown that UV exposure can result in a decline in *F*_V_/*F*_M_. Interestingly, some authors assign this to the effect of UV B [52, 79]. In contrast, other authors conclude that it is UV A that directly affects PS II and causes inhibitory stress [18, 76], which could also be the case in this study, as we used a predominant UV A radiation treatment. UV exposure generated similar declines in *Tetracystis* sp. and *Chlamydomonas nivalis*, but the initial *F*_V_/*F*_M_ values were reached again after an overnight recovery [58].

All three investigated strains contained a considerable amount of antheraxanthin and zeaxanthin. The highest deepoxidation state of the xanthophyll cycle was found in strain G, which goes along with the highest levels of phenolics in control cells of this strain, which could as well be a consequence of other stress factors, already suppressing this strain prior to UV exposure. This argumentation would further be supported by the finding that strain G had the lowest values of *F*_V_/*F*_M_ in control cells. In the other strains, the deepoxidation was nearly 30 % in strain B. This strain shows some signs of light stress, as the xanthophyll cycle pool size and the deepoxidation state increased; however, this was the only strain where UV treatment did not affect *F*_V_/*F*_M_ values significantly. The plastidal pigments were investigated, as quantitative changes could point towards intracellular stress events, e.g. chlorophyll degeneration or increase of xanthophyll cycle pigments. Moreover, with the same HPLC protocol, the presence or absence of any secondary carotenoids (e.g. astaxanthin) was tested and found to be negative. Xiong et al. [79] reported slight degradation of chlorophyll in UV B-sensitive green algal species. Such degradation products occurred in samples of *Zygnema* strain B and strain G in the present study.

Other compounds may be involved in UV protection of *Zygnema* as well. No secondary carotenoids were detected, and MAAs have not been found in other Zygnematophyceae so far [60]. However, other ways of protection than producing intracellular compounds may play a role, and it has been suggested that extracellular structures help to screen UV (e.g. sporopollenin in the cell walls) [78]. *Zygnema* is capable of producing this resistant polymer, but its presence so far has been demonstrated only in the cell walls of zygospores [55]. Such putative substances may not have been extracted from the cell walls by our methods. Extracellular mucilage may also protect cells from UV [29]. Remarkably, in our study, the highest amount of mucilage production was observed in strain B. The presence of mucilage can also explain its significantly low amount of overall pigments per dry weight because a large proportion of dry weight was in fact due to extracellular matrix. Finally, another possibility for explaining UV tolerance may be the formation of macroscopic mats in the habitat, which is also typical for *Zygnema* [31]. Mat-forming growth forms provide self-shading and protect cells against excessive irradiation [9, 73].

## Conclusions

Our study showed the accumulation of various phenolic-like compounds by polar, semi-terrestrial-derived *Zygnema*. The use of an HPLC protocol supported a reliable quantification than comparatively unspecific, colorimetric spectrophotometer assays. The phenolics are obviously involved in UV protection as significantly shown by the response of strain E. Even in *Zygnema* sp. strain G, which was severely stressed by the exposure conditions, a comparably high content of phenolics per dry weight was measured. The different strains, however, started under different physiological conditions. The lower optimal quantum yield in control cells of strain G in comparison to strains B and E could have influenced the UV resistance of this strain. It can be taken as a hint for another stress interacting with the UV in this strain, which is supported also by the very high de-epoxidation state. However, it has to be stated that phenolic pigments generally play an important role in stress response of Zygnematophyceae, above all in species of extreme habitats like glacier surfaces [60], high mountain lakes [18] or semi-terrestrial habitats of polar regions as described in this study. In such locations, synthesis of phenolics has a substantial advantage—they are carbohydrates [21], which make their production ‘cheaper’ in comparison to, e.g. MAAs, containing nitrogen [13].

Our study gives evidence for the importance of phenolics in UV protection in *Zygnema*, but lacks a clear-cut correlation between the degree of damage and putative phenolic contents. Strain G shows damage in the ultrastructure and PS II quantum efficiency, but has as high phenolic content as strain E which did not show damage. On the other hand, strain B has the lowest total phenolics content and shows no signs of damage. This could have different reasons: First, it is possible that the applied UV intensities were too low to provoke significant UV damages in the investigated *Zygnema* strains. Second, the different responses to UV stress in the investigated *Zygnema* strains might as well suggest that various other strategies, such as mucilage production, compounds localised in the cell wall or mat-forming growth, might be involved in overall UV protection. This variation also illustrates the value in using more than a single isolate per genus or major taxon in physiological studies. There are great differences in ecophysiological performance among individual strains, and therefore, generalisations based on the investigation of a single strain might be misleading.

Further work is needed to compare the resistance of polar strains with that of algae from temperate or high-altitude regions where UV stress is naturally stronger. Moreover, to our knowledge, the structure of such phenolics occurring in *Zygnema* has not been determined. The isolation and characterisation of these compounds is currently under work. Finally, a detailed knowledge of special adaptation mechanisms in polar organisms may also be of potential use and commercial interest
